# SIRT1 promotes tumor-like invasion of fibroblast-like synoviocytes in rheumatoid arthritis via targeting TIMP1

**DOI:** 10.18632/oncotarget.21628

**Published:** 2017-10-06

**Authors:** Jiangtao Guo, Wei Zhao, Xuqing Cao, Huiying Yang, Juan Ding, Jingbin Ding, Zifang Tan, Xiaoli Ma, Chunfang Hao, Lili Wu, Zhengjuan Ma, Jianjun Xie, Zhijun Wang

**Affiliations:** ^1^ Cancer Hospital of General Hospital, Affiliated Ningxia People’s Hospital, Key Laboratory of Fertility Preservation and Maintenance of Ministry of Education, Ningxia Medical University, Yinchuan, China; ^2^ Ningxia People’s Hospital, Yinchuan, China; ^3^ The Fifth People’s Hospital of Ningxia, Shizuishan, China; ^4^ Pingluo People’s Hospital, Pingluo, China

**Keywords:** rheumatoid arthritis, invasion, fibroblast-like synoviocytes, SIRT1, deacetylation

## Abstract

Suppression of tissue inhibitor of matrix metalloproteinase (TIMP) is associated with the tumor-like invasion of fibroblast-like synoviocytes (FLSs) that occurs during rheumatoid arthritis-related cartilage destruction. Silent information regulator 2 homolog1 (SIRT1), a histone deacetylase, is widely involved in transcriptional regulation, genomic stability, metabolism and DNA repair. Recent studies suggest that SIRT1 may also impact inflammatory response and the proliferation of FLSs in rheumatoid arthritis (RA). However, it is unknown whether SIRT1 has a role in the tumor-like invasion of FLSs in rheumatoid arthritis. Herein we report that SIRT1 contributes to FLS invasion and cartilage destruction via a TIMP1-dependent mechanism. Elevated SIRT1 in RA synovia suppresses TIMP1 expression via deacetylation of TIMP1-associated histones, thereby disrupting the binding of the transcription factor specificity protein 1 (Sp1) to the TIMP1 promoter. In rats with collagen-induced arthritis, depletion of SIRT1 remarkably promoted TIMP1 expression in synovial tissues and ameliorated cartilage destruction. These results describe a new role for SIRT1 and demonstrate its potential value as a therapeutic target for rheumatoid arthritis.

## INTRODUCTION

Rheumatoid arthritis (RA) is an autoimmune disease characterized by hyperplastic and tumor-like invasive synovial tissue, which induces destruction of joint cartilage and bone. As passive responders and imprinted aggressors, fibroblast-like synoviocytes (FLSs) are a key component of the invasive synovia and have a major role in the erosion of joint cartilage and bone [1]. Activation of matrix metalloproteinases (MMPs) immensely contributes to the tumor-like invasion of FLSs in RA [2, 3]. MMP activity is suppressed by tissue inhibitors of MMPs (TIMPs). Thus, inhibition of TIMPs, like MMP activation, may also promote FLS invasion and cartilage destruction.

Silent information regulator 2 homolog1 (SIRT1), a nicotinamide adenine dinucleotide (NAD)-dependent histone deacetylase with post-translational modifications, is involved in the pathogenesis of cancer, metabolic disease, inflammation and arthritis. Expression of SIRT1 in RA synovia has been shown to be induced by TNF-α further to promote proinflammatory cytokine release and inhibit FLSs apoptosis [4]. However, a recent study has shown that SIRT1 could ameliorate synovial inflammation through inhibiting the differentiation of monocytes to macrophages [5]. Moreover, myeloid deletion of SIRT1 inhibited dendritic cell maturation and further alleviated arthritis in collagen-induced arthritis (CIA) mice [6]. In addition, SIRT1 contributed to FLS proliferation and adhesion during RA progression [7].

In a prior study, we confirmed that SIRT1 was overexpressed in both synovial tissue and FLSs from RA patients. In addition, down-regulation of SIRT1 by a lentiviral shRNA significantly weakened the invasion of RA FLSs. Further studies found that the elevated SIRT1 suppressed TIMP1 in RA FLSs. However, the precise mechanisms linking SIRT1, TIMP1 and the tumor-like invasion of RA FLSs remain unclear.

Our work presented herein explored how SIRT1 suppressed TIMP1 by impeding the binding of transcription factor specificity protein 1 (Sp1) to the TIMP1 promoter (pTIMP1) as well as what molecular mechanisms contributed to the tumor-like invasion of RA FLSs. Our findings present a novel potential strategy for the treatment of RA.

## RESULTS

### SIRT1 negatively correlated with TIMP1 in synovial tissue of RA patients

**Figure 1:**
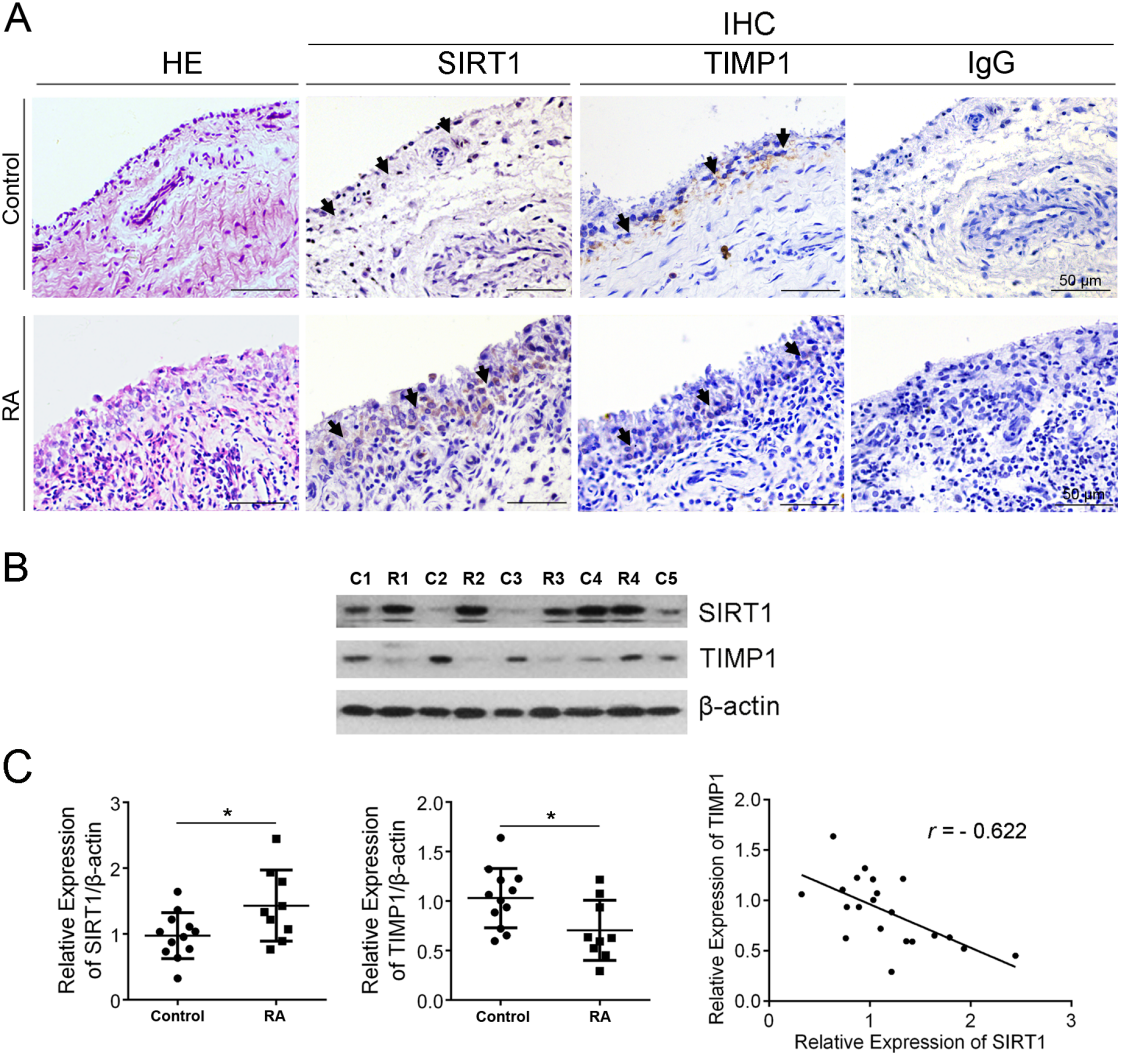
SIRT1 negatively correlated with TIMP1 in synovial tissue of RA patients **(A)** Expression of SIRT1 and TIMP1 in RA and control synovia was investigated using IHC. **(B)** Expression of SIRT1 and TIMP1 in RA (n=10) and control synovia (n=12) was further confirmed using an immunoblot assay. **(C)** Accumulated plots of the immunoblot data show the relative protein expression of SIRT1 and TIMP1. A Pearson’s correlation coefficient was used in the correlative analysis of SIRT1 and TIMP1 expression. The data are represented as mean ± SEM from three independent experiments. *P < 0.05 between the indicated groups.

H&E staining showed hyperplasia and lymphocyte infiltration in RA synovia and a mass of cells with transformed phenotype (large, dark nucleus) in the lining layer of RA synovia (Figure 1A). The levels of SIRT1 and TIMP1 in synovial tissues of patients with RA or with knee joint trauma (control synovia) were measured by immunohistochemistry (IHC). IHC analysis hinted that increased SIRT1 and decreased TIMP1 presented in the lining layer of RA synovia, whereas in the control group, it’s completely the opposite (Figure 1A). Further, immunoblot results confirmed increased SIRT1 and decreased TIMP1 in RA synovia (Figure 1B). As shown in Figure 1C, SIRT1 in RA synovia was significantly higher than in control synovia (*P* < 0.05). Whereas, TIMP1 was lower in RA synovia than in control synovia (*P* < 0.05). Both in RA and control synovia, SIRT1 and TIMP1 had a negative correlation (*r* = -0.622).

### SIRT1 contributed to the invasion of RA FLSs by suppressing TIMP1

**Figure 2:**
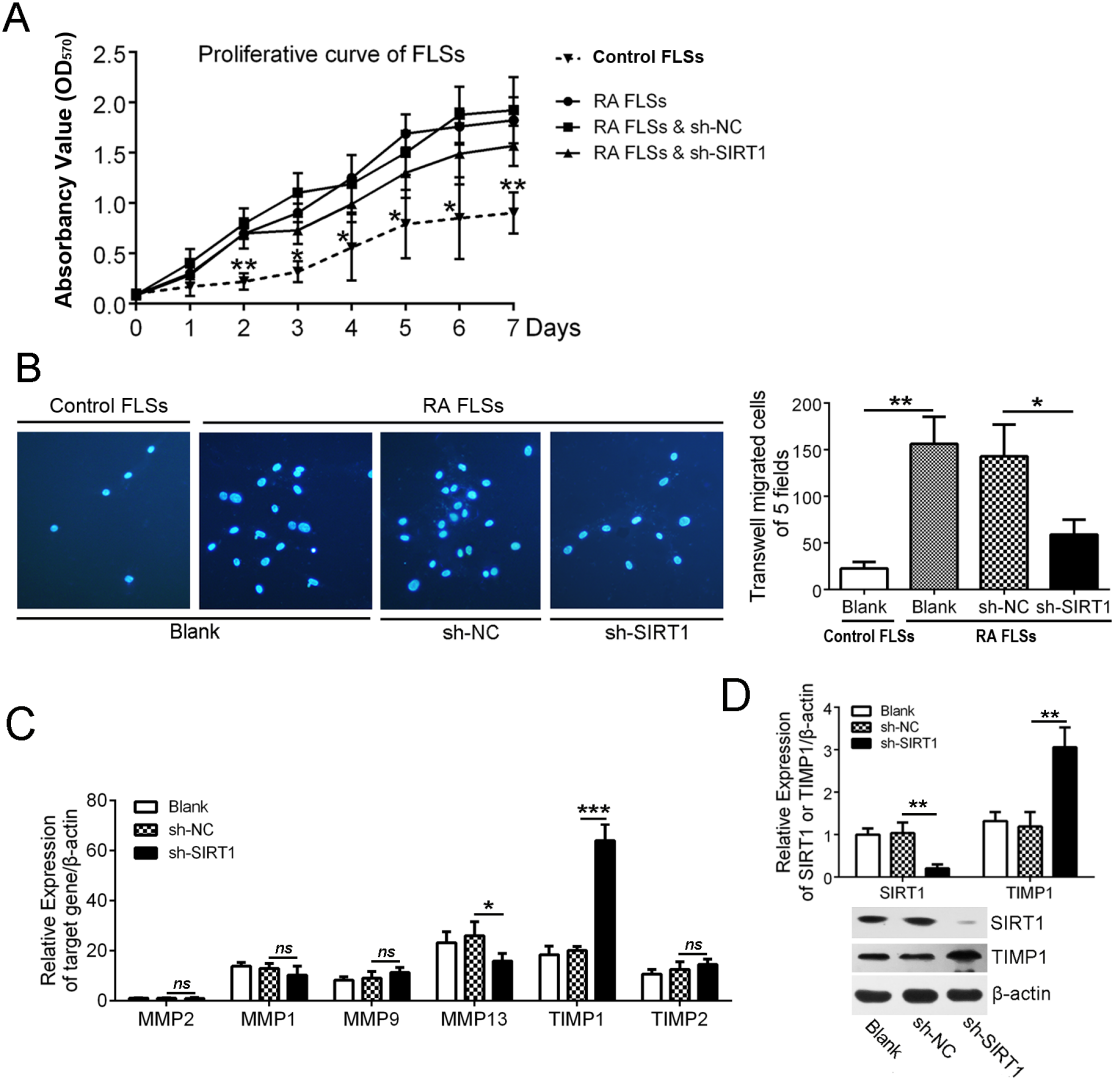
SIRT1 contributed to the invasion of RA FLSs by suppressing TIMP1 **(A)** MTT assay was used to investigate the proliferation capacity of control FLSs and RA FLSs with or without sh-SIRT1 treatment. **(B)** The effect of SIRT1 on the invasion of RA FLSs was assessed using a transwell assay. **(C)** qPCR data shows that the effect of SIRT1 on the relative mRNA expression of MMP1, MMP2, MMP9, MMP13, TIMP1 and TIMP2 in RA FLSs. **(D)** The effect of SIRT1 on TIMP1 expression was confirmed by an immunoblot assay. ^*ns*^ > 0.05, *P < 0.05, **P < 0.01, ***P < 0.001 between the indicated groups. The data are representative of three independent experiments.

To investigate the potential role of SIRT1 in RA synovia, we studied the effect of SIRT1 on proliferation and invasion of FLSs. A lentiviral shRNA of SIRT1 (sh-SIRT1) was used to down-regulate SIRT1 expression in RA FLSs (Figure 2D). As shown in Figure 2A, RA FLSs had a higher speed of proliferation than control FLSs (from synovial tissues with knee joint trauma), but down-regulating SIRT1 had no significant effect on proliferation of RA FLSs. In a transwell assay, RA FLSs showed an increased invasion versus control FLSs. Sh-SIRT1 decreased the invasion of RA FLSs versus vehicle (sh-NC) (Figure 2B). That meant that SIRT1 was necessary for the invasive ability of RA FLSs. Furthermore, the effects of SIRT1 on associated proteins in FLSs were detected using a real-time PCR assay. The results showed that SIRT1 could inhibit TIMP1 expression in RA FLSs and that suppressing SIRT1 allowed for the recovery of TIMP1 expression (Figure 2C). An immunoblot assay confirmed the results of the real-time PCR assay (Figure 2D).

### SIRT1 promotes polymerization of the TIMP1 gene and deacetylated histones and further obstructs transcription factor Sp1 from binding to the TIMP1 promoter

**Figure 3:**
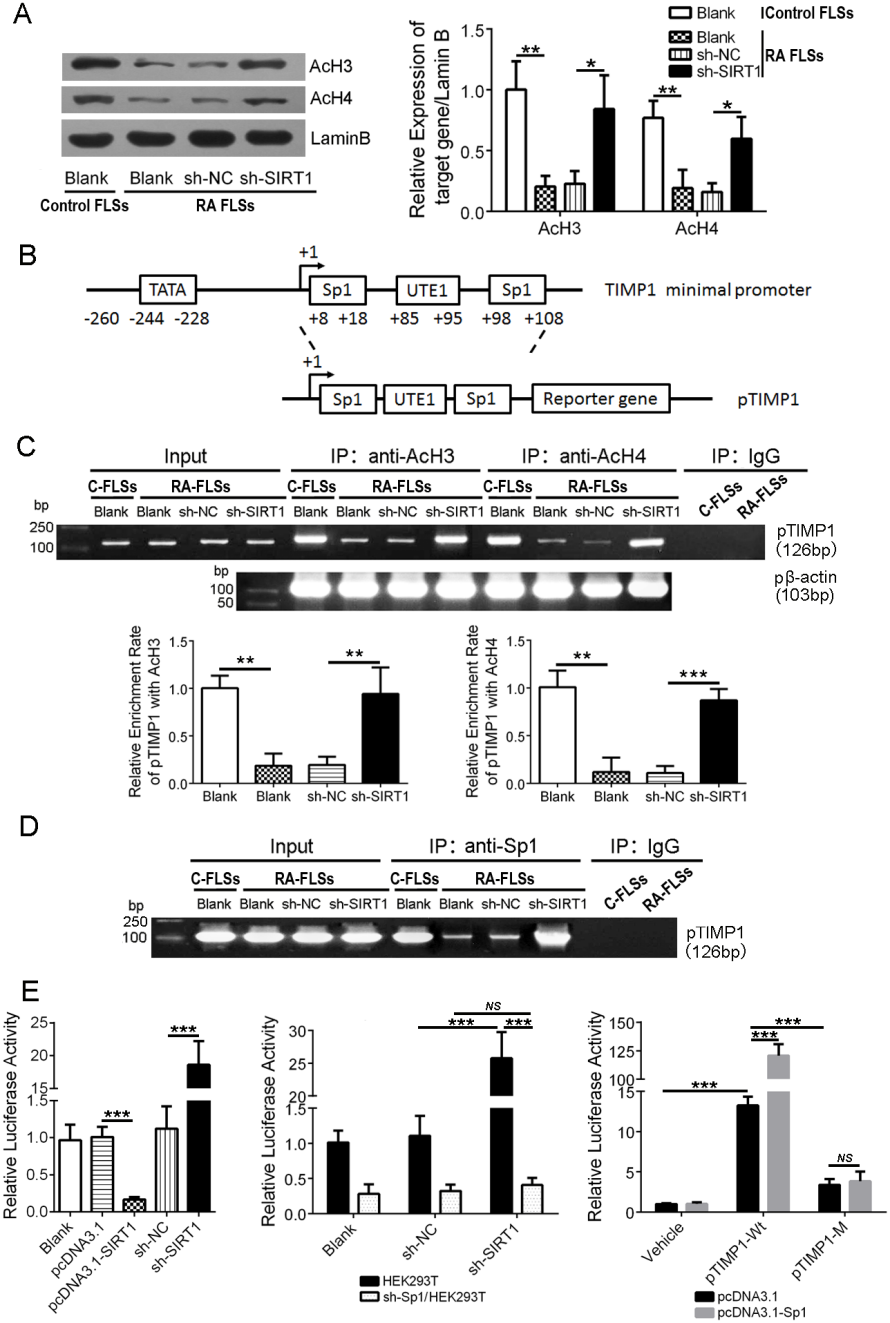
SIRT1 promotes polymerization of the TIMP1 gene and deacetylated histones and obstructs transcription factor Sp1 from binding to the TIMP1 promoter **(A)** Immunoblot analysis of AcH3 and AcH4 expression in FLSs. **(B)** Schematic diagram of TIMP1 promoter (pTIMP1) containing two Sp1 response elements. **(C)** Copies of pTIMP1 directly bound by AcH3 and AcH4 were examined by a Chip assay. The β-actin promoter (pβ-actin) was used to normalize the copies. **(D)** Direct binding of Sp1 to pTIMP1 in control FLSs and in RA FLSs and the effect of SIRT1 on the direct binding activity were examined by a Chip assay. **(E)** A luciferase reporter gene assay was performed to determine the relative activity of pTIMP1 in different cell types: 1) HEK293T cells with up-regulating or down-regulating SIRT1 treatment (left); 2) down-regulating SIRT1 expression in HEK293T cells with or without Sp1 (sh-Sp1/HEK293T) (middle); and 3) pTIMP1-Wt or pTIMP1-M-transfected HEK293T cells without SIRT1 (sh-SIRT1/HEK293T). ^*ns*^ > 0.05, *P < 0.05, **P < 0.01, ***P < 0.001 between the indicated groups. The data are representative of three independent experiments.

SIRT1 is a well-known histone deacetylase. We investigated the expression of acetyl histone H3 (AcH3) and acetyl histone H4 (AcH4) in RA FLSs using an immunoblot assay. As shown in Figure 3A, expression of AcH3 and AcH4 in RA FLSs was lower than in control FLSs (*P* < 0.01), and down-regulating SIRT1 in RA FLSs increased AcH3 and AcH4 levels (*P* < 0.05). To further investigate the effect of SIRT1-mediated histone deacetylation on the transcription activity of TIMP1 promoter, we analyzed the DNA sequence of the TIMP1 promoter (pTIMP1). As shown in Figure 3B, there were two putative response elements of transcription factor Sp1 within the pTIMP1. The primers covering pTIMP1 were used for the CHIP assays. The results of AcH3 and AcH4 antibody immunoprecipitation (IP) showed that a 5.4-fold (or 8.4-fold) decrease in pTIMP1 binding to AcH3 (or AcH4) in RA FLSs compared with control FLSs. Down-regulating SIRT1 in RA FLSs significantly increased the copies of pTIMP1 binding-AcH3 (or AcH4) (*P* < 0.01) (Figure 3C). The results of the other CHIP assay with Sp1 antibody revealed that Sp1 could directly bind to pTIMP1 (Figure 3D). Moreover, Sp1 antibody pulled down more pTIMP1 DNA in control FLSs than in RA FLSs (*P* < 0.01) and decreasing SIRT1 expression in RA FLSs could increase the copies of pTIMP1 binding Sp1 (Figure 3D). A luciferase reporter gene system was used to confirm the regulation of Sp1 on TIMP1 transcription. As shown in Figure 3E, down-regulation of SIRT1 in HEK293T cells enhanced the activity of pTIMP1 but not in Sp1 shRNA-treated HEK293T cells. In addition, overexpression of Sp1 in HEK293T cells without SIRT1 enhanced the activity of pTIMP1 wild type (pTIMP1-Wt) but not pTIMP1 mutant (pTIMP1-M) (Sp1 response elements mutant). These results demonstrate that histone deacetylation by SIRT1 could weaken the affinity between the transcription factor Sp1 and TIMP promoter and further suppress TIMP1 gene transcription in RA FLSs.

### Depletion of SIRT1 significantly relieved cartilage erosion in CIA rats

**Figure 4:**
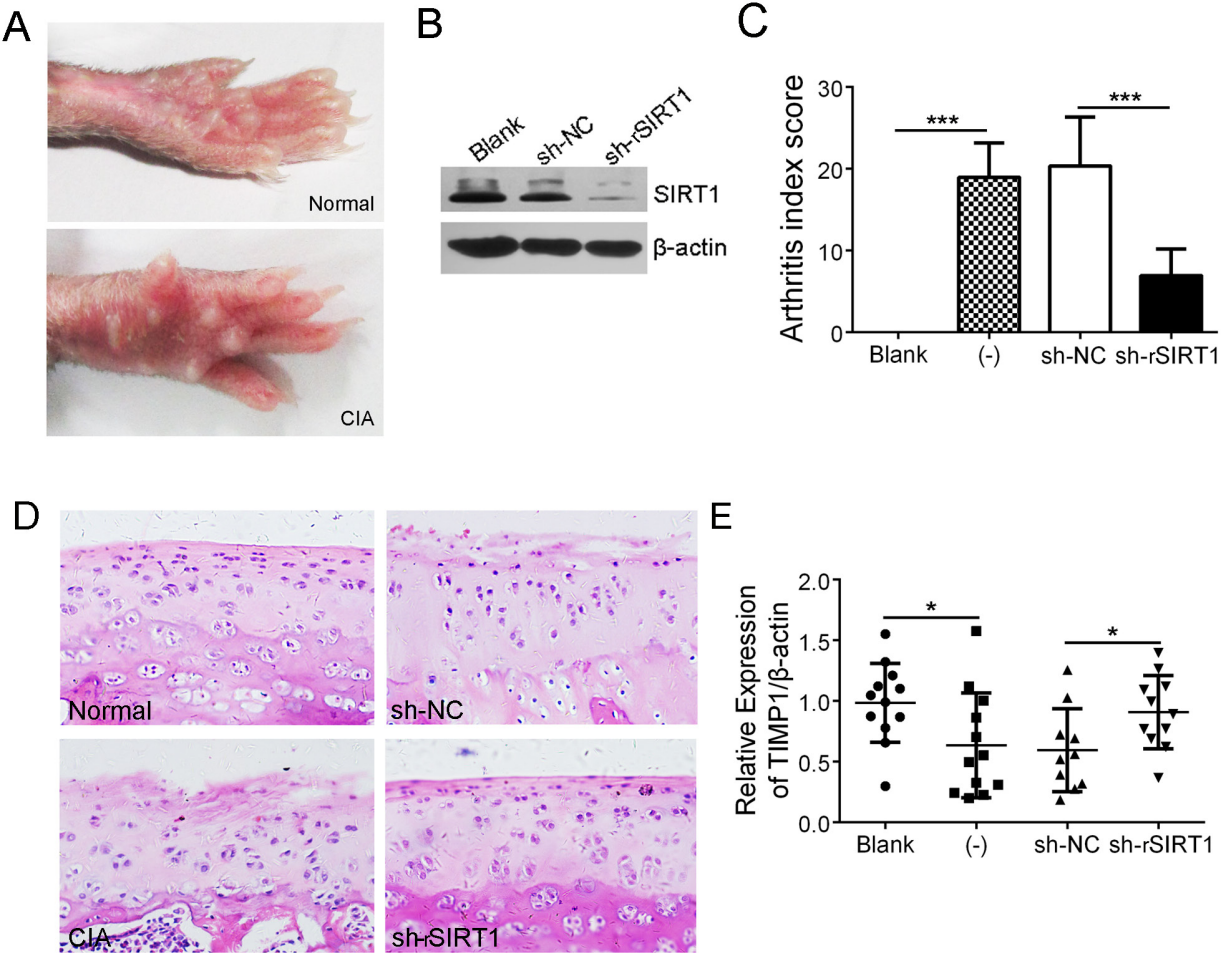
Depletion of SIRT1 significantly relieved cartilage erosion in CIA rats **(A)** Macroscopic performance of arthritis, such as swelling, was observed in the CIA rats. **(B)** Interference efficiency of a lentiviral shRNA of rSIRT1 (sh-rSIRT1) or its control lentivirus (sh-NC) in synovia of rats was measured by immunoblot assay. **(C)** The arthritis index scores in normal (n = 12), CIA (-) (n = 12), CIA with sh-NC treatment (n =10) and CIA with sh-rSIRT1 treatment groups (n =11) were counted and contrasted. **(D)** Hind limbs from rats were sectioned and stained with H&E. Original magnification 200×. **(E)** Expression of TIMP1 in synovia was analyzed using real-time PCR. *P < 0.05, **P < 0.01, ***P < 0.001 between the indicated groups. The data are representative of three independent experiments.

The hind paws of CIA rats showed obvious swelling (Figure 4A). A lentiviral shRNA of rattus SIRT1 (sh-rSIRT1) was used to interfere with SIRT1 expression *in vivo*. Immunoblot analysis showed that the injection of sh-rSIRT1 significantly depleted SIRT1 in the synovial tissue of rats versus control (Figure 4B). As shown in Figure 4C, sh-rSIRT1 reduced the arthritis index score (AIS) of CIA rats. H&E-stained sections from the knee joint showed that sh-rSIRT1 prevented articular cartilage destruction in CIA rats, whereas, non-treated CIA rats showed perichondrium erosion and cartilage destruction (Figure 4D). Further, real-time PCR analysis revealed that CIA rats expressed less TIMP1 in knee joint synovia than normal rats, but down-regulating SIRT1 in synovia of CIA rats promoted more TIMP1 expression (Figure 4E). These data suggest that depletion of SIRT1 relieved the articular cartilage erosion in CIA rats.

## DISCUSSION

Fibroblast-like synoviocytes (FLSs), unique cells that ubiquitously distribute the intimal layer of the joint synovia, play a critical role in maintaining the structural and functional integrity of joints by regulating the renewal of the cartilage and subchondral bone and the secretion of the synovial fluid. In rheumatoid arthritis (RA), FLSs display surprisingly tumor-like behavior, including increasing in number, mediating inflammation, and acquiring aggressive phenotypes, all of which contribute to joint cartilage erosion [1].

Previous reports have shown that plenty abnormal signaling pathways are involved in the transformation of FLSs from friend to foe, such as excessive proliferation and subdued apoptosis of FLSs via P53 [8, 9], P21 [10–12], Ras [13], Myc [14]. Further pathway examples include glucose-6-phosphate isomerase (G6PI) [15], HIF-1α [16] and TNFα [17], which mediate hypoxia-induced angiogenesis, contributed to pannus formation in RA synovia. Furthermore, plenty of activated signaling pathways promote the secretion of inflammatory factors and aggravated immune response [18, 19]. Particularly, regulators of extracellular matrix (ECM), such as MMPs [20, 21], which enhanced the invasiveness of FLSs, are regarded as the “killer” of joint destruction. However, the activity of MMPs is controlled by TIMPs. Thus, down-regulation of TIMPs contributes to improving FLS invasion and cartilage destruction as well [22, 23].

Silent information regulator 2 homolog1 (SIRT1), a NAD-dependent histone deacetylase, is involved in the pathogenesis of cancer [24], metabolic disease [25], inflammation [26] and arthritis [27]. The roles of SIRT1 in RA are mainly focused on the immune response [4–6], the proliferation and adhesion of RA FLSs [7]. To the best of our knowledge, there are no reports concerning the effect of SIRT1 on FLS invasion and joint destruction.

The present study reports that SIRT1 is overexpressed in either synovial tissue or FLSs from RA patients. Elevated SIRT1 suppresses TIMP1 expression in RA FLSs, whereas, down-regulating SIRT1 significantly weakens RA FLS invasion. However, the precise mechanisms linking SIRT1, TIMP1 and tumor-like invading FLSs in RA remains unclear.

Histone acetylation and deacetylation of a gene’s promoter region is a pair of the crucial factors regulating the gene’s transcriptional activity [28]. First, we analyzed the DNA sequence of the TIMP1 promoter (pTIMP1). Its minimal promoter region contains two response elements bound by transcription factor Sp1. The present study further shows that SIRT1, a histone deacetylase, down-regulates histone acetylation in the TIMP1 minimal promoter region and restrains the chromatin structure of the TIMP1 gene from depolymerizing. The tight chromatin structure in the TIMP1 gene hinders the binding of Sp1 to the TIMP1 promoter. In contrast, depletion of SIRT1 could suppress histone deacetylation and, thus, open the chromatin structure of the TIMP1 gene and promote the binding of Sp1 to the TIMP promoter. Similarly, *Y. Zhao et al.* also demonstrated that SAHA, a histone deacetylase inhibitor, promotes MICA expression via inhibiting deacetylation of MICA-associated histones [29]. Although these results *in vitro* demonstrated that SIRT1 was a crucial factor in TIMP1-mediated invasion of RA FLSs, we still performed experiments in animals. In our studies, a collagen-induced arthritis (CIA) model of SD rats was used to investigate the role of SIRT1 in synovia invasion and cartilage destruction in RA pathogenesis. Down-regulation of SIRT1 expression by lentiviral shRNA significantly decreased the arthritis index score and alleviated joint cartilage of CIA rats.

In conclusion, our findings are the first to focus on the role of SIRT1 on tumor-like invasion of the synovia and cartilage destruction in RA pathogenesis. Moreover, we reveal the epigenetic mechanism of SIRT1 suppressing TIMP1 expression via deacetylating TIMP1-assoicated histones to hinder transcription factor Sp1 binding to the TIMP1 promoter. Our findings suggest that SIRT1 may be a potentially valuable therapeutic target for RA.

## MATERIALS AND REAGENTS

### Tissues, cell lines and reagents

Synovial tissue was from patients with RA or with knee joint trauma (control synovia) in the General Hospital or the Affiliated Ningxia People’s Hospital of Ningxia Medical University. All of the procedures were approved by the Ningxia Medical University Ethics Committee. RA FLSs and control FLSs were purchased from Cell Applications, Inc. HEK293T cell line was purchased from Cell Bank of Chinese Academy of Sciences. Homo spices SIRT1 shRNA lentiviral particles (sh-SIRT1), Sp1 shRNA lentiviral particles (sh-Sp1), Scramble shRNA lentiviral particles (sh-NC) and rattus SIRT1 shRNA lentivirus (sh-rSIRT1) were purchased from Santa Cruz Biotechnology, Inc. HEK293T cells were infected by sh-SIRT1 or sh-Sp1. Stable clones expressing the shRNA via puromycin (Santa Cruz Biotechnology, Inc.) were selected to prepare Sh-Sp1/HEK293T cells and sh-SIRT1/HEK293T cells.

### Plasmid construction

As the template for the PCR assay, plasmid pECE-hSIRT1 (Addgene) was used to amplify the cDNA of SIRT1, which was inserted into a vector pcDNA3.1(+) and a vector pCDH to construct pcDNA3.1(+)-SIRT1. The synthesized TIMP1 minimal promoter DNA (pTIMP1-Wt), its mutant (pTIMP1-M) and gene β-actin promoter DNA (pβ-actin) (Sangon Biotech) were respectively inserted into a vector pGL3-basic to clone pGL3-pTIMP1-Wt, pGL3-pTIMP1-M and pGL3-pβ-actin.

### RNA isolation and real-time PCR

For reverse transcription-PCR, total RNA was extracted from RA FLSs using TRIzol reagent (Beyotime), and then cDNA was prepared using an EasyScript Reverse Transcriptase (Transgen Biotech), following the manufacturer’s instructions. Real-time PCR was performed in an 7500 Fast Real-time PCR system (ThermoFisher) and SYBR *Premix Ex Taq*™ II (TaKaRa) under the following conditions: denaturation at 95°C for 30s, followed by 40 cycles of 95°C for 3s, 60°C for 30s. The relative expression of target gene was normalized to β-actin mRNA. Every experiment was performed in triplicate. The sequences of primers are as follows:

SIRT1 5’-GCGGGAATCCAAAGGATAAT-3’/5’-CTGTTGCAAAGGAACCATGA-3’

TIMP1 5’-CATGGAAAGCCTCTGTGGAT-3’/5’-AAGAAGCTGCAGGCATTGAT-3’

MMP1 5’-CCCTCTTGAACTCACATGTTATG-3’/5’-ACTTTCCTCCCCTTATGGATTCC-3’

MMP2 5’-GGCCTCTCCTGACATTGACCTT-3’/5’-GGCCTCGTATACCGCATCAATC-3’

MMP9 5’- TTTGACAGCGACAAGAAGTGG-3’/5’-AGGGCGAGGACCATAGAGG-3’

MMP13 5’- CTGCCTTCCTCTTCTTGAG-3’/5’-TGCTGCATTCTCCTTCAGGA-3’

TIMP2 5’- CTACGGAAGATCTCAATAGCG-3’/5’-GGGACTCTCAATCCTCGTC-3’

β-actin 5’-CTGTCCACCTTCCAGCAGATGT-3’/5’-CGCAACTAAGTCATAGT CCGCC-3’

### Immunoblot analysis

Total proteins were extracted from synovial tissues or FLSs using a radioimmunoprecipitation assay (RIPA) lysis buffer (Beyotime). Protein concentrations were determined by a BCA protein assay kit. Equal amounts of protein (50μg) were separated using SDS-PAGE electrophoresis and transferred to PVDF membranes as previously described [21]. The blots were subsequently probed with SIRT1 mAb (ThermoFisher), TIMP1 mAb (ThermoFisher), Sp1 mAb (Santa Cruz Biotechnology, Inc.) or β-actin mAb (Santa Cruz Biotechnology, Inc.) and HRP-conjugated secondary antibodies, and then were developed using an enhanced chemiluminescence (ECL) reagent (ThermoFisher).

### MTT cell proliferation assay

FLS proliferation was evaluated using a 3-(4, 5-Methylthiazol-2-yl)-2, 5-diphenyl-tetrazo-lium bromide (MTT) assay kit (Beyotime, China). Briefly, control FLSs and RA FLSs with different treatments were seeded in 96-well plates. According to the manufacturer’s protocol, MTT solution and Formazan solution were added in turn, and the optical densities were measured at 570 nm. All experiments were performed in triplicate.

### Transwell invasion assay

RA FLSs cultured in 5% FBS-DMEM medium were infected by sh-SIRT1 (or sh-NC), in turn, were seeded into the upper chamber of a transwell insert (Costar) coated with Matrigel (Sigma), and the lower chamber contained 10% FBS-DMEM medium. After incubation at 37°C for 24h, none-migratory cells on the upper surface were cleaned. The migrated cells were stained with DAPI and were counted (five fields/well) under a fluorescence microscope.

### Dual-luciferase reporter assay

The activity of the TIMP1 promoter (pTIMP1) was analyzed using a luciferase reporter assay. The plasmids pcDNA3.1-Sp1 or pcDNA3.1, together with pGL3-pTIMP1 and phRL-TK, were co-transfected into HEK293 cells, and then the activities of *Firefly* luciferase (*FLuc*) and *Renilla* luciferase (*RLuc*) were measured 24h later according to the manufacturer’s protocol (Promega) as previously described [21].

### Chromatin immunoprecipitation (CHIP)

CHIP assay was performed according to the manufacturer’s protocol (Millipore). Briefly, control FLSs and RA FLSs infected with sh-SIRT1 or sh-NC were fixed with 1% formaldehyde for 10 min at room temperature. Then, nuclear extract was re-suspended in lysis buffer and was sonicated to prepare DNA fragments. AcH3 antibody, AcH4 antibody, Sp1 antibody (Santa Cruz Biotechnology, Inc.) or control IgG was respectively used for immunoprecipitation. The DNA fragments were amplified using the pTIMP1 primers (5’-ATCTCCTTTCGTCGGCC-3’/5’-AATGTCCACGCTAGG-3’) or pβ-actin primers (5’-AGTTGCCTTTTATGGCTCG-3’/5’-CGAG CCATAAAAGGCAACT-3’).

### Establishment and treatment of a CIA rat model

Specific pathogen-free (SPF) Sprague-Dawley (SD) rats obtained from the Lab Animal Center of Ningxia Medical University. The protocol was approved by the Animal Ethics Committee. Forty eight-week-old male SD rats were randomly divided into 4 groups: Blank, CIA, CIA with sh-NC and CIA with sh-rSIRT1, as previously described [21]. All CIA rats were immunized using a subcutaneous injection with a collagen emulsion (Sigma) on their tail roots. After 3 weeks, the rats were performed a secondary immunity on their left hind paws. Sh-rSIRT1 or sh-NC was injected into the left knee joint cavity of the CIA rats on Day 3 after the secondary immunization and the injection was repeated three times each ten days.

### Histopathological analyses

Joint tissues of rats were fixed, decalcified and embedded. Sections from paraffin tissue blocks were performed a hematoxylin and eosin (H&E) staining according to standard protocols and were observed the symptoms of synovia proliferation, inflammatory cell infiltration and cartilage destruction under a microscope, which were used to evaluate the severity of arthritis. In immunohischemical assay, paraffin sections which were de-waxed, rehydrated and underwent antigen retrieval successively were detected with SIRT1 or TIMP1 antibody (ThermoFisher) to confirm their expression and distribution under a microscope (Olympus).

### Statistical analysis

All data are presented as the mean ± SD in three independent experiments and were analyzed with SPSS 13.0. Statistical significance was calculated by one-way ANOVA analysis of variance between two groups of the LSD method. Pearson correlation coefficient was performed to correlation analyses between SIRT1 and TIMP1. *P* values less than 0.05 were considered statistically significant.
